# Performance of single-use syringe versus multi-use MR contrast injectors: a prospective comparative study

**DOI:** 10.1038/s41598-020-60697-w

**Published:** 2020-03-03

**Authors:** F. Struik, J. J. Futterer, W. M. Prokop

**Affiliations:** Department of Radiology and Nuclear Medicine, Radboudumc, Nijmegen, The Netherlands

**Keywords:** Health care, Medical research

## Abstract

The goal of this study was to compare performance parameters of a single-use syringe and a multi-use MR contrast injector. We compared preparation time, cost for disposables and volumes of contrast material used for a single-use (SI) and a multi-use (MI) MR contrast injector in a prospective cross-over trial. During the first study period all consecutive patients eligible for dynamic MR on two systems were included during a period of 20 working days. After 10 days, the injector was switched. Radiographer satisfaction was evaluated using a questionnaire. Contrast usage and waste on system MI was optimised by extra instructions for our radiographers and measured during the second study period of 10 consecutive working days. A total of 202 and 163 patients for systems SI and MI were included, respectively. Average preparation time was 4:55 min for SI and 2:24 min for MI (p < 0.05). Contrast waste for SI was 13% using 7.5 ml syringes. Contrast waste for MI was 5% for 7.5 ml containers. Costs for disposables were lower for MI if more than 5 patients per day were injected. Radiographer satisfaction was higher for MI (4.7 versus 2.8 on a 5-point scale; p < 0.05). The multi-use MR contrast injector led to higher radiographer satisfaction, shorter preparation time, and lower cost if more than 5 patients were injected per day. In addition, cheaper contrast containers of 15 or 30 ml could be used for the first patients if more than 2 or more than 4 injections are performed per day, potentially leading to lower contrast waste.

## Introduction

Contrast enhanced (CE) magnetic resonance (MR) imaging has been shown to contribute between 30% and 45% of MR examination volume in clinical practice^1^. Power injectors were developed for injection of contrast media (CM) in body MR and allow for specific flow rates for uniform vascular and visceral enhancement^2^.

The costs of an injection of MR contrast are dependent on several factors: preparation and setup time, contrast usage and waste and costs of disposables. Most hospitals use single-use syringe systems, which have to be separately prepared for each exam. Shortening or reducing any of the steps associated with CM administration may influence operational efficiency^3–5^. Since their introduction, gadolinium based contrast agents are injected by a fixed dose, rather than a fixed volume^6^. In several body area’s a dose of 0.1 mmol/kg has shown to be adequate^7–11^. When using a pre-filled syringe, only a fixed injected volume can be used - the contents of the syringe - and the excess has to be disposed of at the end of each exam. This results in a variable amount of waste.

More recently, multi-use MR contrast injectors were developed. These injectors offer the option to use larger bottles instead of syringes, injecting multiple patients form the same bottle. This could potentially give less waste and preserving the option of individual dosing. The injector is prepared and contrast bottles are loaded ahead of program. At the start of each exam only a tubing set is changed, potentially saving time at the start of each individual exam.

The purpose of our prospective comparative study was to compare a novel multi-use MR contrast injector, compared to a single-use injector to standard syringe-base single-use injector in terms of efficiency, radiographer satisfaction, and costs of disposables.

## Materials and Methods

### Study design and patient selection

This was a single-center, prospective, comparative cohort study. The study was approved by our institutional review board.

#### Patients

During the first part of the study, all patients scheduled for CE MR during 4 consecutive weeks where included. The second part of the study took place during 2 consecutive weeks, also including all patents scheduled for CE MR. An intravenous access suitable for power injection had to be available. Patients < 18 years or with an intravenous access not suitable for power injection were excluded.

#### MR injector

The contrast media injections were performed either by a single-use syringe injector (SI, single injector) or multi-use injector (MI, multi-use injector). The total amount of contrast material (gadobutrol 1 mmol/ml; Gadovist; Bayer) and its injection rate for each examination were dependent on the patient’s body weight, the requested examination type, and the status of the intravenous access.

System SI, a single-use syringe injector (Mallinckrodt Optistar LE injector, Liebel-Flarsheim Company), has two syringes, one for holding 7.5 ml contrast material and one for holding a 25 ml saline flush. A y-shaped tube system is used to connect the two syringes to the intravenous access. At the end of each exam any unused CM was disposed. The amount of discarded CM was recorded.

System MI, a multi-use MR contrast injector (Max 3 power injector, Ulrich Medical). This contrast injector allows simultaneous loading of up to two CM bottles of a volume varying from 10 ml to 100 ml on each media slot. The contrast bottles could be containing the same contrast agent or different ones, for example, a routine Gadolinium compound and a liver-specific one. This facilitates multiple injections without the need to re-load CM or saline in between patients. This injector uses one daily cassette approved for 24 hour use^12^. Sterile connector tubing including two check valves is available for each patient. At the end of the day, any unused CM was disposed of. The amount of discarded CM was recorded.

Both the contrast power injectors (single- and multi-injector) were CE-approved, which means that the medical device, including device safety, complies with the applicable EU regulations. Hygienic issues are covered in the design of both injectors. The SI-injector requires a new syringe and set of disposables for every patient; the MI-injector a daily cassette and a set of disposables for each patient.

#### Study setup

In order to get a mixed case load, patients were selected on two MR systems, a 1.5 T and a 3 T MR scanner (Avanto and Skyra, Siemens Healthineers). The radiographers started with a four week training period, to make sure that they were all familiarised with both injector systems.

After this training period, the first part of the study started and took place during 20 consecutive working days. During the first 10 consecutive working days, all patient scheduled for dynamic or non-dynamic CE MR were injected using the multi-use MR contrast injector (system MI).

**Figure 1:**
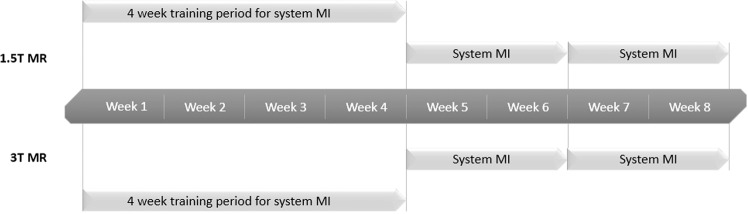
Timeline of study setup of phase 1 at both 1.5 and 3 T MR scanner.

During the second 10 consecutive working days all patient scheduled for CEMR were either injected with system SI in case of a dynamic exam or injected manually in case of a non-dynamic exam, as in our routine workflow. **(**Fig. 1**)**.

After this first part we analysed our contrast usage and waste, and optimised this by extra instructions for our radiographers.

Then we started the second part of the study, which took place during 10 consecutive working days. Again, all patient scheduled for dynamic or non-dynamic CE MR were injected using the multi-use MR contrast injector (system MI). We recorded the amount of contrast used and contrast waste per scanner per day.

All experiments were performed in accordance with relevant guidelines and regulations. The study was approved by our institutional review board, and written informed consent was waived.

### Quantitative assessment

Contrast-enhanced MR was performed as per our department protocol for the requested indication. Our trained MR radiographers operated the 2 MR scanners. A team of observers recorded the data regarding the performance of the power injectors on a predesigned template for the study duration.

The observers recorded patient-related information such as type of MR scan and planned examination time. Information related to power injector use was also recorded: preparation or loading time, time to connect the system to the patient, volume of CM used and the CM waste (calculated as the CM loading volume minus the CM injected) for each patient. We calculated the optimal amount of contrast that could be used, and the minimal amount of waste per day.

#### Costs of disposables

Costs of disposables are variable and depending on local factors. We therefore calculated the average cost of disposables per patient with CE MR, normalizing these costs to the standard system at our institution (system SI). The average costs of disposables for system SI are consistent, containing one set of tubing per exam. These were compared with the costs of system MI, which consist of the costs of one cassette per day and the costs of one set of patient tubing per exam. Results were plotted in a graph.

### Qualitative assessment

Qualitative evaluation of the power injectors included a survey of the radiographer satisfaction after the end of the 20 day study period. A 5-point scale was used to capture their satisfaction with both power injectors for time-efficiency, user friendliness and their overall opinion.

### Statistical analysis

A Student t test was used for evaluation of the quantitative data. The Mann-Whitney U test was performed in analysis of the qualitative data with ordinal level of measurement. A *p* value less than 0.05 was considered to indicate a significant difference. Descriptive statistical analysis was also performed. All statistical analysis was performed using Social science statistics^13^.

## Results

### Phase 1

In phase 1, we scanned 202 patients with system SI, using 1376 ml of contrast. For system MI, we included all 163 patients, using 1159 ml of contrast. There were no statistically significant differences between the numbers of scanned patients and amount of contrast that was used per patient. The average total time used per exam was 4:55 min for system SI and 2:24 min for system MI (p < 0.05), respectively **(**Table 1**)**.

**Table 1:** Time efficiency of system SI versus system MI.

	System SI	System MI	
Daily setup	0 min	5:09 min per day	
		0:38 min per exam	
Pre-exam	2:29 min	0 min	
Installing patient	2:21 min	1:34 min	
Cleaning up	~0:05 min	1:35 min per day	
		0:12 min per exam	
Total per exam	4:55 min	2:24 min	**p** < **0,05**

Contrast waste was 13% using system SI, which uses 7.5 ml syringes. Contrast waste was 17% using 30 ml bottles with system MI. We calculated that system MI would have had contrast waste of 8% in case 15 ml and 30 ml bottles would have been available and 5% in case 7.5 ml, 15 ml and 30 ml would have been available (p < 0,05).

### Phase 2

In phase 2 we used a total amount of 2115 ml of contrast with 224 ml contrast waste (11%). The average amount of contrast used daily schedule was 71 ml, varying from 30 to 150 ml per programme per day. Contrast waste was 8 ml per daily schedule, varying from 0 to 21 ml.

### Costs of disposables

**Figure 2:**
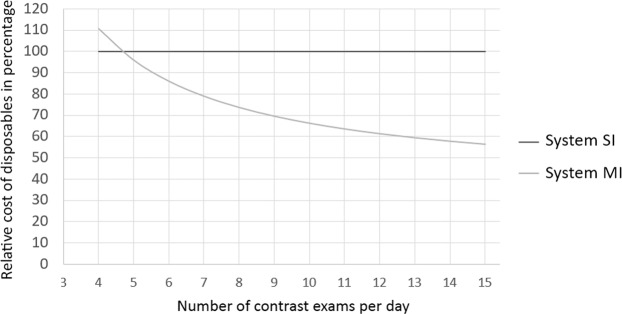
Percentage of costs of disposables per contrast exam versus system SI. Cost of system SI normalised at 100%, using one set of tubing per exam. System MI uses one daily cassette, costing 297% compared tot system SI and one set of disposables per exam, costing 36%.

The costs of disposables for system SI consist of one set of tubing per exam, with no extra daily costs. The costs of disposables used by system MI consist of one set of tubing per exam and one cassette per day. When the total costs per day are divided by the number of contrast exams per day, the ratio of the costs are declining. At 5 exams per day, the costs are similar, above this system MI gets relatively cheaper (Fig. 2).We scanned 8 patient on average per day, varying from 5 to 14 a day.

### Radiographer questionnaire

#### Time efficiency

System SI was considered easier to set up at the beginning of the day than system B (4.4 vs 3.7; p = 0.04). Preparation per patient was significantly easier for system MI (SI 3.5 – MI 4.7; p < 0.01).

Cleaning at the end of the day was significantly easier for system SI (4.9 vs 4.1; p < 0,01).

There were no significant differences in ease of connecting to the patient (SI 4.6-MI4.8; p = 0.24) and cleaning at the end of the exam (SI4.6-MI4.8; p = 0.29). (Table 2**)**.

**Table 2:** Results of the radiographer questionnaire.

Time efficiency	System SI	System MI	p value
Preparation per day	4.4	3.7	0.04
Preparation per patient	3.8	4.7	<0.01
Connecting to the patient	4.6	4.8	0.24
Cleaning at the end of the exam	4.6	4.8	0.29
Cleaning at the end of the day	4.9	4.1	<0.01
**User-friendliness**			
Use of display	4.1	4.1	0.81
Setting of parameters	3.5	4.2	0.07
Correction of parameters	4	3.4	0.06
Overall user-friendliness	3.7	4.4	0.01
**Overall opinion**			
Time efficiency	2.4	4.7	<0.01
Patient-friendliness	4	4.3	0.36
Is this system suitable to use in normal daily practise	2.8	4.7	<0.01

#### User friendliness

There were no significant differences on ease of use of display (SI 4.1 – MI 4.1; p = 0.81), setting of parameters (SI 3.5-MI 4.2; p = 0.07), and correcting of parameters in case of an error (SI 4.0- MI3.4 p = 0.06). The overall user-friendliness system MI was considered more user-friendly then SI scoring 3.7 versus 4.1 (p = 0.01).

#### Overall opinion and preferences

System MI was considered overall more time efficient (SI 2.4-MI 4.7 p < 0.01) and more suitable to be used in normal daily practise (SI 2.8-MI 4.7; p < 0.01). There was no significant difference in overall patient-friendliness (SI 4-MI 4,7 p = 0.36). 94% of radiographers preferred system MI over system SI (p < 0.01).

## Discussion

Our study identified the advantages of using a multi-use MR contrast versus a single use contrast injector during CE MR exams scans. We showed that use of the multi-use MR contrast injector results in a shorter preparation time of contrast, lower costs of disposables and higher radiographer’s satisfaction. The MI system also offers the option to use cheaper contrast bottles instead of syringes.

Several studies have been conducted comparing performance parameters of CT power injectors in terms of work flow and time efficiency; contrast use, contrast waste and operating costs^3,14–16^. Optimizing these performance factors in MR can be expected to result in lower costs and increase patient throughput. The multi-use MR contrast injector was found to be more time-efficient, with an average total contrast administration time per exam of 2:24 min compared to 4:55 min for the single use injector, saving 2:31 min per exam. This decrease in contrast administration time can be used to increase the total number of exams possible per day or to accommodate emergency exams more flexibly.

The overall process of administering a IV contrast with the multi-use MR contrast injector was considered easier and quicker and was preferred by the vast majority of radiographers in our practice despite the fact that the radiographers were more familiar with the single use MR contrast injector the time of the study because of its routine use in our institution for many years.

The multi-use MR contrast injector decreased the costs of disposables if more than 5 patients are scanned per day. With an average number of 8 patients scanned with contrast per day at the scanners included in this study, we found a 26% lower cost of disposables. Since costs of disposables depend on a variety of factors (e.g., currency rates, local price arrangements), absolute costs cannot be transferred from one institution to the other. The exact effect of these time savings on a daily MR schedule will be dependent on local factors, such as total time per exam and working hours^4^.

The expected savings in contrast due to reduced contrast waste was not found at our institution because we only had 30 ml bottles of contrast agent available at the time of the study. If such a bottle had to be used towards the end of the day, a substantial amount of waste occurred. This could have been avoided with the use of smaller bottle sizes towards the end of the scan day. In order to reach optimal contrast efficiency it is necessary to estimate the amount of contrast to be used ahead of the program. Another factor that might have contributed to this is our patient population with a lot of complex and ill patients (for example ICU patients), and exams duration varying from 30 to 90 minutes. We expect that the expected savings can be reached in an outpatient setting with more ambulant patient and shorter scan duration. Since the daily cassette that contains the tubing of the contrast pump is approved to be used for 24 hours, all tubing including the contrast bottles has to be disposed of after this period. Any amount of contrast that is not used has to be discarded, even if one of the containers has been prepared less than 24 hours before. This potentially can lead to high contrast waste. This might be optimised if the approval could be extended beyond 24 hours, so that the residual contrast could be used at the beginning of the next daily program.

A limitation in our study is that it was carried out in a single institution and the results of our study may be not directly applicable to other hospital settings. For example local variation in daily MR schedules containing contrast and non-contrast enhanced exams, as well as dynamic contrast enhanced exams. For this reason we provided estimates of relative costs for the two systems depending on the number of contrast-enhanced exams per day. We used relative cost instead of absolute cost to better correct for influences such as currency effects and local price arrangements.

In conclusion, we found that use of the multi-use MR contrast injector led to higher time efficiency with a shorter preparation time of contrast, lower costs of disposables if more than 5 patients are scanned per day and higher radiographer’s satisfaction. The system also offer the option to use bigger bottles of contrast instead of syringes, potentially lowering contrast waste below 5%.

### Ethical approval

The contrast power injectors were both CE-approved devices which are routinely used in clinical practice. For our study, we compared both power injectors which are routinely used in clinical practice. This comparative study was approved by the institutional review board of the CMO region Arnhem-Nijmegen, The Netherlands, and written informed consent was waived (according to GCP guidelines).
